# Supplementary material for: A programmable qudit-based quantum processor
Source: Nat Commun. 2022 Mar 4;13:1166. doi: 10.1038/s41467-022-28767-x (PMC8897515; doi:10.1038/s41467-022-28767-x)
Supplement: Supplementary file 1 — Supplementary Information [file 41467_2022_28767_MOESM1_ESM.pdf]

# Supplementary Information: A Programmable Qudit-based Quantum Processor

Yulin Chi[1,⋆], Jieshan Huang[1,⋆], Zhanchuan Zhang[1,⋆], Jun Mao[1], Zinan Zhou[1],Xiaojiong Chen[1], Chonghao Zhai[1], Jueming Bao[1], Tianxiang Dai[1], Huihong Yuan[1,2], Ming Zhang[3], Daoxin Dai[3], Bo Tang[4], Yan Yang[4], Zhihua Li[4], Yunhong Ding[5,6], Leif K. Oxenløwe[5,6], Mark G. Thompson[7], Jeremy L. O'Brien[8], Yan Li[1,9,10,11], Qihuang Gong[1,2,9,10,11], Jianwei Wang[1,2,9,10,11†]

[1] *State Key Laboratory for Mesoscopic Physics, School of Physics, Peking University, Beijing, 100871, China*

[2] *Beijing Academy of Quantum Information Sciences, Beijing 100193, China*

[3] *State Key Laboratory for Modern Optical Instrumentation, College of Optical Science and Engineering, Ningbo Research Institute, International Research Center for Advanced Photonics, Zhejiang University, Hangzhou 310058, China*

[4] *Institute of Microelectronics, Chinese Academy of Sciences, Beijing 100029, China*

[5] *Department of Photonics Engineering, Technical University of Denmark, 2800 Kgs. Lyngby, Denmark*

[6] *Center for Silicon Photonics for Optical Communication (SPOC), Technical University of Denmark, 2800 Kgs. Lyngby, Denmark*

[7] *Quantum Engineering Technology Labs, H. H. Wills Physics Laboratory and Department of Electrical and Electronic Engineering, University of Bristol, BS8 1FD, Bristol, United Kingdom*

[8] *Department of Physics, The University of Western Australia, Perth 6009, Australia*

[9] *Frontiers Science Center for Nano-optoelectronics & Collaborative Innovation Center of Quantum Matter, Peking University, Beijing, 100871, China*

[10] *Collaborative Innovation Center of Extreme Optics, Shanxi University, Taiyuan 030006, Shanxi, China*

[11] *Peking University Yangtze Delta Institute of Optoelectronics, Nantong 226010, Jiangsu, China.*

⋆ *These authors contributed equally to this work.*

† *Emails: jww@pku.edu.cn*

## Supplementary Note 1: Device fabrication and experimental setup

The qudit-based quantum processor ($d$-QPU) was fabricated on the silicon-photonic platform, using the complementary metal-oxide-semiconductor processes. A thin layer of photoresist was first to spin on an 8 inches silicon-on-insulator (SOI) wafer and it was followed by the 248 nm deep ultraviolet (DUV) photolithography processes to define the circuit patterns. The patterns in the soft mask were transferred to the silicon layer by the inductively coupled plasma (ICP) etching processes. Two etching-depth waveguides were adopted in our chip, i.e., deeply (shallowly) etched ones with an etching depth of 220nm (70nm). A layer of 1μm-thick silicon dioxide ($SiO_2$) was deposited by plasma-enhanced chemical vapor deposition (PECVD). Finally, a layer of 50 nm-thick titanium nitride (TiN) was deposited on top of waveguides to form thermal-optical phase-shifters (TOPS) (see resistance with a mean of $361(2)\Omega$ in Fig.2c). Photon-pairs were generated in silicon waveguides with a 450 nm width, 220 nm height, and 1.2 cm length, by the SFWM nonlinear process. Multimode interferometers (MMIs) with a width of 2.8 μm and length of 27 μm were used as balanced beamsplitters with a 0.2dB loss. The well balanced MMIs allows high-quality interference with a measured mean visibility of 0.992(16), as shown in Fig.2c. The diagram and optical microscopy image of the $d$-QPU are shown in Fig.2d. The chip was optically coupled via single-mode optical fibers using grating couplers with a loss of 4dB per coupler. A continuous wave laser at the wavelength of 1550.12nm, which was amplified to 100mW power by an erbium-doped fiber amplifier (EDFA) and spectrally purified by a dense wavelength division multiplexing (DWDM) module, was used to excite the array of on-chip SFWM sources. In our experiment, we chose the signal photons at 1545.32nm and idler photons at 1554.94nm, respectively. In order to separate and locally operate the signal-idler photons, their wavelength difference was set to be half of the free spectrum range (FSR) of the on-chip asymmetric MZIs that was measured to be 18.2nm. The $d$-QPU integrates multidimensional entangled sources, arbitrary single-qudit logic gates, arbitrary two-qudit multi-value controlled logic gates, and arbitrary single-qudit projectors. It can be reprogrammed and

reconfigured to implement different quaternary algorithms. Single-photons were routed off-chip for detection, by an array of fiber-coupled superconducting nanowire single-photon detectors (SNSPDs) with an averaged efficiency of 85%. Residual pump photons were removed from the single photons by DWDMs. Photon coincidence counts were recorded by a multichannel time interval analyzer (TIA). In the typical setting of our experiment, we obtained the two-photon coincidence rate to be ∼kHz (can be further improved to ∼100 kHz using a higher power excitation), which is 6 orders higher than the four-photon rate of ∼mHz in a device with the same size of Hilbert space as the two-ququarts device here[58]. Note that, the comparison of photon detection rate should take the consideration the performance and loss of the quantum devices as well as their pumping and measurement apparatuses. The detection rate is dependent on the photon-pair generation rate (probability, efficiency) of the parametric sources which relies on the pump power and also the source designs (e.g, microrings or waveguides), loss of on-chip optical components (e.g, chip-fiber coupler, MZI, beamsplitter, waveguide crosser, and loss of optical waveguide), and loss of off-chip apparatuses for the measurement and detection of single-photon states (e.g, single-photon detector, optical filter). That is being said, a comprehensive comparison of photon detection rate requires much more experimental details. In our experiment, we implemented different integration time of data collections in different parts of experiments, for examples, 10s/measurement for quantum state tomography, 5s/measurement for quantum process tomography, 20s/measurement for the Deutsch-Jozsa and Bernstein-Vazirani algorithms, and 20s/measurement for the d-ary IPEA and order finding algorithms. The chip was wired-bounded (Fig.2d) on a printed circuit board (PCB) and all TOPSs were individually controlled by a multichannel electronic controller with a 16-bit resolution. A classical processor was used to control qudit states and process experimental outcomes of the $d$-QPU. A classical-quantum hybrid setup was adopted for the implementations of Kitaev's quantum phase estimation and order-finding in quaternary.

## Supplementary Note 2: Definitions of qudit states and logic gates

The generalised quantum fourier transform gate $\mathcal{F}_d$ and generalised Hadamard gate $\mathcal{H}_d$ on a single qudit are defined as:

$$\begin{cases} \mathcal{F}_d \left|k_i\right\rangle = \frac{1}{\sqrt{d}} \sum_{j=0}^{d-1} \omega^{ij} \left|j\right\rangle = \left|f_i\right\rangle \\ \mathcal{H}_d \left|k_i\right\rangle = \frac{1}{\sqrt{d}} \sum_{j=0}^{d-1} h_{i,j} \left|j\right\rangle = \left|h_i\right\rangle \end{cases} \tag{2}$$

where $\omega = e^{\mathrm{i}2\pi/d}$; $d$ refers to the local size of qudit; $|k_i\rangle$ is the computational basis; $|h_i\rangle$ is the Hadamard basis and $h_{i,j} = (-1)^{i\odot j}$, where $i \odot j = \oplus_{m=1}^{d-1} i_m j_m$ denotes the bitwise inner product of $i$ and $j$ expressed in the binary representation.

The generalised Pauli $\mathcal{X}_d$ and $\mathcal{Z}_d$ gate on a single qudit are defined as:

$$\begin{cases} \mathcal{Z}_d \left|k_i\right\rangle = \omega^i \left|k_i\right\rangle \\ \mathcal{X}_d \left|k_i\right\rangle = \left|k_i \oplus_d 1\right\rangle \end{cases} \tag{3}$$

where $\oplus_d$ is addition module of $d$.

Quantum devices implementing unitary transformation can be efficiently characterised by the complementary classical fidelity, which are measured in two complementary basis of the unitary[62]. We adopted the complementary basis for the measurements of the truth tables (see results in Fig 3) as below: computational basis: $|k_i\rangle$; Fourier basis: $|f_i\rangle = \mathcal{F}_4 |k_i\rangle$; Hadamard basis: $|h_i\rangle = \mathcal{H}_4 |k_i\rangle$; the basis $|l_i\rangle$, another eigenstate of the $\mathcal{F}_4$ gate:

$$\begin{cases} \left|l_0\right\rangle = \frac{1}{2}(-\left|0\right\rangle + \left|1\right\rangle + \left|2\right\rangle + \left|3\right\rangle) \\ \left|l_1\right\rangle = \frac{1}{\sqrt{2}}(\left|0\right\rangle + \left|2\right\rangle) \\ \left|l_2\right\rangle = \frac{1}{2}(\left|0\right\rangle + \left|1\right\rangle - \left|2\right\rangle + \left|3\right\rangle) \\ \left|l_3\right\rangle = \frac{1}{\sqrt{2}}(\left|1\right\rangle - \left|3\right\rangle) \end{cases} \tag{4}$$

the basis $|a_i\rangle = \frac{1}{2}\sum_{j=0}^{3} h_{i,j} |l_j\rangle$; the basis $|b_i\rangle = \mathcal{H}_4 |a_i\rangle$.

## Supplementary Note 3: The scheme of $n$-photon $d$-dimensional multi-qudit quantum processor

We firstly take the two-ququart $d$-QPU device in Fig.2c as an example to explain the quantum state evolution process, and then discuss its generalisation to the multiquidt $d$-QPU device, as shown in Fig.2a. Firstly, an array of four integrated SFWM photon sources produces a four-level entangled bell state, using the non-degenerate SFWM process, and the state then can be written in the Fock basis as $(|2000\rangle + |0200\rangle + |0020\rangle + |0002\rangle)/2$. Having the deterministic separation of two single photons with different colours at an array of four asymmetric MZIs that demultiplex the two photons, the state evolves into an eight modes state as $(|10001000\rangle + |01000100\rangle + |00100010\rangle + |00010001\rangle)/2$ (see

stage I in Fig.2c). Note $|0\rangle$ refers to the vacuum state, and $|1\rangle$, $|2\rangle$ present the number of photons in their spatial modes. The first 4 dits indicate the signal photon and the last 4 dits indicate the idler.

When rewriting the Fock state of each photon into the logical state using the following mapping:

$$\begin{cases} |0001\rangle_{\text{Fock}} \leftrightarrow |0\rangle_{\text{logical}} \\ |0010\rangle_{\text{Fock}} \leftrightarrow |1\rangle_{\text{logical}} \\ |0100\rangle_{\text{Fock}} \leftrightarrow |2\rangle_{\text{logical}} \\ |1000\rangle_{\text{Fock}} \leftrightarrow |3\rangle_{\text{logical}} \end{cases} \tag{5}$$

it results in a four-level generalised Bell state of $|\text{Bell}\rangle_4$ as:

$$|\text{Bell}\rangle_4 = \frac{|0\rangle_1|0\rangle_2 + |1\rangle_1|1\rangle_2 + |2\rangle_1|2\rangle_2 + |3\rangle_1|3\rangle_2}{2}, \tag{6}$$

where the subscripts of the logical state $|i\rangle_{1,2}, i = 0,1,2,3$ denote the two ququarts. The first and second ququarts are encoded in the signal and idler photons, respectively. We then expand each mode of the idler photon into a 4-dimensional space, in total 16-dimensional space in the stage II (see Fig.2c), which is equivalent to the space of two-ququart states. We define each nearest 4-mode as a "layer", and then define a third ququart to represent the layer information. The logical state of the third ququart is encoded in the 4 layers, i.e, $|j\rangle_3, j = 0,1,2,3$, as shown in Supplementary Fig.1, where the subscript denotes the third qudit. Within the layer, the four modes form the second qudit and allows an arbitrary single-qudit preparation of $|\phi\rangle_2$. In short, the space expansion process induces an additional qudit state 3, and the state can be derived as:

$$|\psi\rangle_a = \frac{|0\rangle_1|\phi\rangle_2|0\rangle_3 + |1\rangle_1|\phi\rangle_2|1\rangle_3 + |2\rangle_1|\phi\rangle_2|2\rangle_3 + |3\rangle_1|\phi\rangle_2|3\rangle_3}{2}, \tag{7}$$

where the third ququart presents the layer (or process) information in stage II (their coherence is retained), and the subscripts denote the qudits 1,2,3. The stage IV allows an arbitrary single-qudit local operation of $\mathcal{O}$ on the second qudit, we thus obtain the state as:

$$|\psi\rangle_b = \frac{1}{\sqrt{d}} \sum_{i=0}^{d-1} (|k_i\rangle_1 \otimes \mathcal{O}_i |\phi\rangle_2 \otimes |k_i\rangle_3), (d = 4), \tag{8}$$

where the operation $\mathcal{O}_i$ is locally performed on the qudit 2 state $|\phi\rangle_2$. We performed a sequence of Hadamard operations $\{\mathcal{H}_2^{0,1}, \mathcal{H}_2^{2,3}, \mathcal{H}_2^{1,2}\}$ on the qudit 3 as shown in the stage V in Fig.2c (physically it is realised by cascaded MMIs, up to some $Z$-phases which can be compensated by the phase-shifters in the qudit projectors), where the subscripts refer to the dimensionality of Hadamard gates and the superscripts refer to their modes. By coherently compressing the 16-dimensional space back into the 4-dimensional space, it results in a state as:

$$\begin{aligned} |\psi\rangle_c = & \frac{|0\rangle_1 \otimes \mathcal{O}_0 |\phi\rangle_2 + |1\rangle_1 \otimes \mathcal{O}_1 |\phi\rangle_2}{2\sqrt{2}} |0\rangle_3 \\ & + \frac{|0\rangle_1 \otimes \mathcal{O}_0 |\phi\rangle_2 - |1\rangle_1 \otimes \mathcal{O}_1 |\phi\rangle_2 + |2\rangle_1 \otimes \mathcal{O}_2 |\phi\rangle_2 + |3\rangle_1 \otimes \mathcal{O}_3 |\phi\rangle_2}{4} |1\rangle_3 \\ & + \frac{|0\rangle_1 \otimes \mathcal{O}_0 |\phi\rangle_2 - |1\rangle_1 \otimes \mathcal{O}_1 |\phi\rangle_2 - |2\rangle_1 \otimes \mathcal{O}_2 |\phi\rangle_2 - |3\rangle_1 \otimes \mathcal{O}_3 |\phi\rangle_2}{4} |2\rangle_3 \\ & + \frac{|2\rangle_1 \otimes \mathcal{O}_2 |\phi\rangle_2 - |3\rangle_1 \otimes \mathcal{O}_3 |\phi\rangle_2}{2\sqrt{2}} |3\rangle_3. \end{aligned} \tag{9}$$

In our experiment, as shown in Fig.2c, we used the detection of the qudit 3 at the second mode, i.e, $|1\rangle_3$, so as to return the $\mathcal{MVCU}_d$ gate. The $Z$-phases before the operations $\mathcal{O}_i$ are compensated by phase-shifters in the qudit measurement stages. Before we discuss the success probability of the $\mathcal{MVCU}_d$ gate in a general $n$ photon $d$-dimensional device, we first discuss the two-ququart device operated by a general 4-dimensional Hadamard gate in the Stage V. And our experimental implementations have the same success probability by multiplexing the repeated measurements. We now consider a case: replacing the cascaded 2-dimensional Hadamard gates of $\{\mathcal{H}_2^{0,1}, \mathcal{H}_2^{2,3}, \mathcal{H}_2^{1,2}\}$ by a general 4-dimensional Hadamard gate (which requires more MMIs at all outputs of the stage V), as shown in Supplementary Figure 1. It returns a

state as:

$$
\begin{aligned}
|\psi\rangle_c' = & \frac{|0\rangle_1 \otimes \mathcal{O}_0 |\phi\rangle_2 + |1\rangle_1 \otimes \mathcal{O}_1 |\phi\rangle_2 + |2\rangle_1 \otimes \mathcal{O}_2 |\phi\rangle_2 + |3\rangle_1 \otimes \mathcal{O}_3 |\phi\rangle_2}{4} |0\rangle_3 \\
& + \frac{|0\rangle_1 \otimes \mathcal{O}_0 |\phi\rangle_2 - |1\rangle_1 \otimes \mathcal{O}_1 |\phi\rangle_2 + |2\rangle_1 \otimes \mathcal{O}_2 |\phi\rangle_2 - |3\rangle_1 \otimes \mathcal{O}_3 |\phi\rangle_2}{4} |1\rangle_3 \\
& + \frac{|0\rangle_1 \otimes \mathcal{O}_0 |\phi\rangle_2 + |1\rangle_1 \otimes \mathcal{O}_1 |\phi\rangle_2 - |2\rangle_1 \otimes \mathcal{O}_2 |\phi\rangle_2 - |3\rangle_1 \otimes \mathcal{O}_3 |\phi\rangle_2}{4} |2\rangle_3 \\
& + \frac{|0\rangle_1 \otimes \mathcal{O}_0 |\phi\rangle_2 - |1\rangle_1 \otimes \mathcal{O}_1 |\phi\rangle_2 - |2\rangle_1 \otimes \mathcal{O}_2 |\phi\rangle_2 + |3\rangle_1 \otimes \mathcal{O}_3 |\phi\rangle_2}{4} |3\rangle_3 .
\end{aligned} \tag{10}
$$

We rewrite the qudit 1 state in the Hadamard basis as:

$$
\begin{aligned}
|\psi\rangle_c' = & \frac{|h_0\rangle_1 \otimes (\mathcal{O}_0 |\phi\rangle_2 + \mathcal{O}_1 |\phi\rangle_2 + \mathcal{O}_2 |\phi\rangle_2 + \mathcal{O}_3 |\phi\rangle_2) + |h_1\rangle_1 \otimes (\mathcal{O}_0 |\phi\rangle_2 - \mathcal{O}_1 |\phi\rangle_2 + \mathcal{O}_2 |\phi\rangle_2 - \mathcal{O}_3 |\phi\rangle_2)}{8} |0\rangle_3 \\
& + \frac{|h_2\rangle_1 \otimes (\mathcal{O}_0 |\phi\rangle_2 + \mathcal{O}_1 |\phi\rangle_2 - \mathcal{O}_2 |\phi\rangle_2 - \mathcal{O}_3 |\phi\rangle_2) + |h_3\rangle_1 \otimes (\mathcal{O}_0 |\phi\rangle_2 - \mathcal{O}_1 |\phi\rangle_2 - \mathcal{O}_2 |\phi\rangle_2 + \mathcal{O}_3 |\phi\rangle_2)}{8} |0\rangle_3 \\
& + \frac{|h_0\rangle_1 \otimes (\mathcal{O}_0 |\phi\rangle_2 - \mathcal{O}_1 |\phi\rangle_2 + \mathcal{O}_2 |\phi\rangle_2 - \mathcal{O}_3 |\phi\rangle_2) + |h_1\rangle_1 \otimes (\mathcal{O}_0 |\phi\rangle_2 + \mathcal{O}_1 |\phi\rangle_2 + \mathcal{O}_2 |\phi\rangle_2 + \mathcal{O}_3 |\phi\rangle_2)}{8} |1\rangle_3 \\
& + \frac{|h_2\rangle_1 \otimes (\mathcal{O}_0 |\phi\rangle_2 - \mathcal{O}_1 |\phi\rangle_2 - \mathcal{O}_2 |\phi\rangle_2 + \mathcal{O}_3 |\phi\rangle_2) + |h_3\rangle_1 \otimes (\mathcal{O}_0 |\phi\rangle_2 + \mathcal{O}_1 |\phi\rangle_2 - \mathcal{O}_2 |\phi\rangle_2 - \mathcal{O}_3 |\phi\rangle_2)}{8} |1\rangle_3 \\
& + \frac{|h_0\rangle_1 \otimes (\mathcal{O}_0 |\phi\rangle_2 + \mathcal{O}_1 |\phi\rangle_2 - \mathcal{O}_2 |\phi\rangle_2 - \mathcal{O}_3 |\phi\rangle_2) + |h_1\rangle_1 \otimes (\mathcal{O}_0 |\phi\rangle_2 - \mathcal{O}_1 |\phi\rangle_2 - \mathcal{O}_2 |\phi\rangle_2 + \mathcal{O}_3 |\phi\rangle_2)}{8} |2\rangle_3 \\
& + \frac{|h_2\rangle_1 \otimes (\mathcal{O}_0 |\phi\rangle_2 + \mathcal{O}_1 |\phi\rangle_2 + \mathcal{O}_2 |\phi\rangle_2 + \mathcal{O}_3 |\phi\rangle_2) + |h_3\rangle_1 \otimes (\mathcal{O}_0 |\phi\rangle_2 - \mathcal{O}_1 |\phi\rangle_2 + \mathcal{O}_2 |\phi\rangle_2 - \mathcal{O}_3 |\phi\rangle_2)}{8} |2\rangle_3 \\
& + \frac{|h_0\rangle_1 \otimes (\mathcal{O}_0 |\phi\rangle_2 - \mathcal{O}_1 |\phi\rangle_2 - \mathcal{O}_2 |\phi\rangle_2 + \mathcal{O}_3 |\phi\rangle_2) + |h_1\rangle_1 \otimes (\mathcal{O}_0 |\phi\rangle_2 + \mathcal{O}_1 |\phi\rangle_2 - \mathcal{O}_2 |\phi\rangle_2 - \mathcal{O}_3 |\phi\rangle_2)}{8} |3\rangle_3 \\
& + \frac{|h_2\rangle_1 \otimes (\mathcal{O}_0 |\phi\rangle_2 - \mathcal{O}_1 |\phi\rangle_2 + \mathcal{O}_2 |\phi\rangle_2 - \mathcal{O}_3 |\phi\rangle_2) + |h_3\rangle_1 \otimes (\mathcal{O}_0 |\phi\rangle_2 + \mathcal{O}_1 |\phi\rangle_2 + \mathcal{O}_2 |\phi\rangle_2 + \mathcal{O}_3 |\phi\rangle_2)}{8} |3\rangle_3 ,
\end{aligned} \tag{11}
$$

where $|h_i\rangle_1, i = 0, 1, 2, 3$ denotes the four Hadamard basis of the qudit 1. One can notice the joint measurements of the qudits 1 and 3 in the bases of $\{|h_0\rangle_1 \otimes |0\rangle_3$, $|h_1\rangle_1 \otimes |1\rangle_3$, $|h_2\rangle_1 \otimes |2\rangle_3$, $|h_3\rangle_1 \otimes |3\rangle_3\}$, all return the same controlled-unitary operation of $(\mathcal{O}_0 + \mathcal{O}_1 + \mathcal{O}_2 + \mathcal{O}_3)$ on the second qudit state $|\phi\rangle_2$. We here remark a few points: Firstly, the coherent evolution of qudits states and the processing of algorithms require the multiple path interference in the $d$-ary Fourier transform gate to get the desired solution, that corresponds to measurements of the $x$-register ququart states in the Fourier basis (similar for the Hadamard basis, but up to some additional phases that can be compensated). Such coherent operation and readout in the Fourier basis are necessary for the achievement of quantum parallelism, where the evaluations of the function for the multiple inputs are executed in parallel. Secondly, the success probability of realising the above entangling controlled-operation of $(\mathcal{O}_0 + \mathcal{O}_1 + \mathcal{O}_2 + \mathcal{O}_3)$ on the $|\phi\rangle_2$ is 1/4. And, other combination of measurements also work with 1/4 probability. For example, measuring in the bases of $\{|h_1\rangle_1 \otimes |0\rangle_3$, $|h_0\rangle_1 \otimes |1\rangle_3$, $|h_3\rangle_1 \otimes |2\rangle_3$, $|h_2\rangle_1 \otimes |3\rangle_3\}$ all returns the success of $(\mathcal{O}_0 - \mathcal{O}_1 + \mathcal{O}_2 - \mathcal{O}_3)$ on the $|\phi\rangle_2$, which requires a $Z$-phase compensation before the unitary $\mathcal{O}_i$ in the four qudit projectors. The scheme in Supplementary Fig.1a presents a spatial (passive) multiplexing, while in our experiment, we implemented a temporal (passive) multiplexing. We instead fixed the measurement of the third qudit in the $|1\rangle_3$ mode and repeated the measurements of the first qudit in the four Hadamard bases. With correct phase compensations before the unitary $\mathcal{O}_i$ in a single qudit projector, as shown in Fig.2c, we obtain the $\mathcal{MVCU}$ gate with a 1/4 probability. The two approaches are equivalent to each other, in term of the success probability of 1/4. The experimental implementation requires a less number of phase-shifters and classical controls, but at the cost of longer time measurements.

Thus, the process of "space expansion–local operation–coherent compression" results in the multi-level entangling state as $\frac{1}{2}\sum_{i=0}^{3} |k_i\rangle \otimes \mathcal{O}_i |\phi\rangle$, where the $\mathcal{O}_i$ gate is entangled with the $x$-register qudit state $|k_i\rangle$. We obtain the $\mathcal{MVCU}$ gate as

$$
\mathcal{MVCU} |x\rangle |y\rangle := |x\rangle \mathcal{O}^x |y\rangle , \tag{12}
$$

where the $x$-register qudit $|x\rangle$ can be arbitrarily operated by the single-qudit logic in stage III, the $y$-register qudit $|y\rangle$ can be arbitrarily prepared in stage II, and the multi-value controlled $\mathcal{O}^x$ can be arbitrarily operated in stage IV. The two qudit states can be locally measured by arbitrary projectors in stage VI and VII, respectively.

We next show that, for a $n$-photon $d$-dimensional multiqudit $\mathcal{MVCU}$ gate, it also works with a $(1/d)$ success probability regardless of the number of photon $n$. In a $n$-photon $d$-dimensional device, each photon locally evolves into the single-qudit operator $\mathcal{O}_{i,j}, i = \{0, 1, ...n - 1\}, j = \{0, 1, ...d - 1\}$, and the third qudits $|k\rangle_3$ defined in the $d$ layers are operated by a $d$-dimensional Hadamard gate. We obtain a $n$-photon

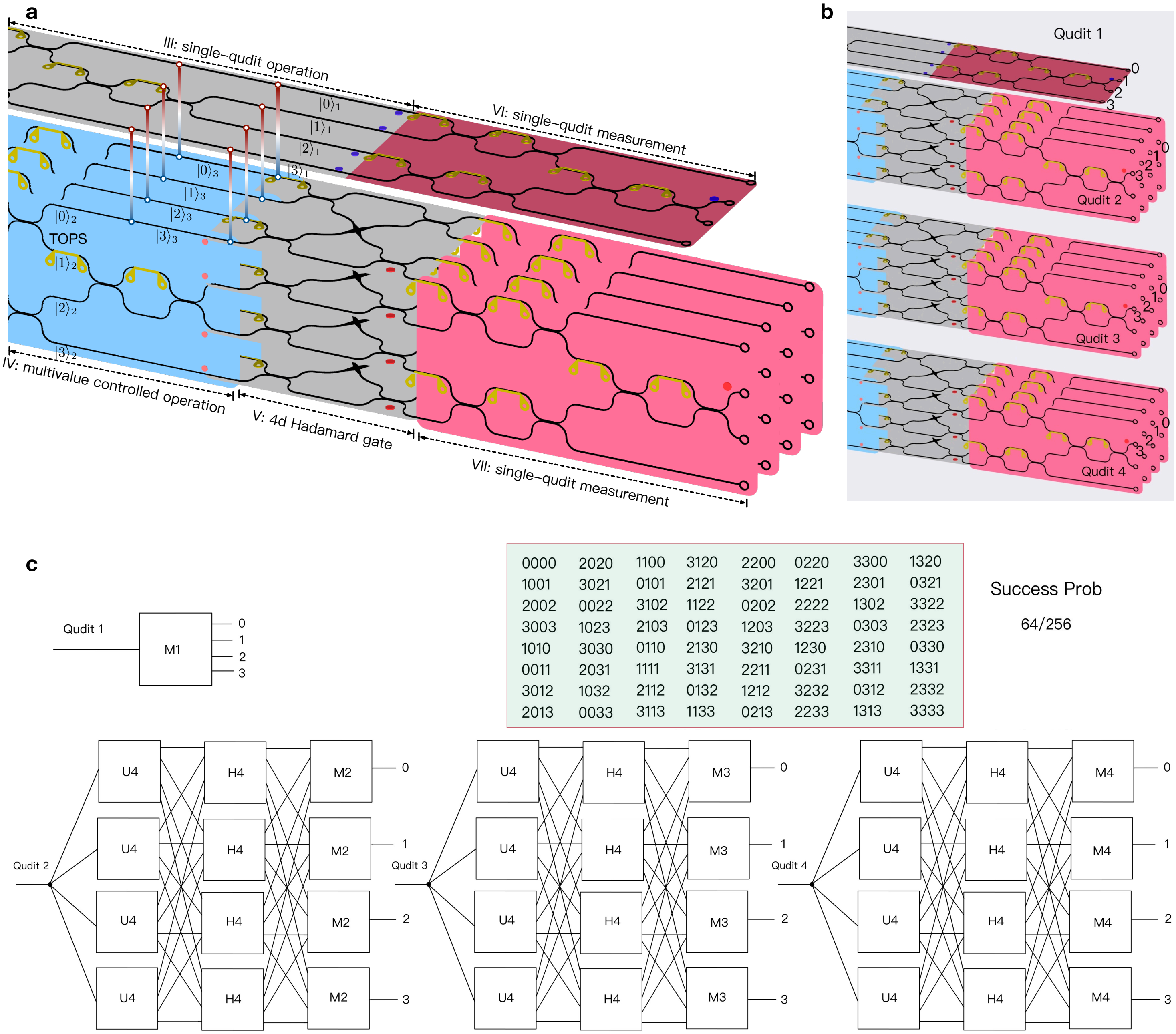


**Supplementary Figure 1: Schemes for $n$-photon $d$-dimensional qudit-based quantum processor. a**, for a two-photon ququart device, by using the general 4-dimensional Hadamard gate, and detecting photons at the all outputs of the compress stage V with a certain pattern (as shown in Eq.(11)), it gives rise to a $1/4$ probability of the ququart $\mathcal{MVCU}$ gate. **b** and **c**, a four-photon ququart device is taken as an example to show the $1/d$ success probability of the four-photon ququart $\mathcal{MVCU}$ gate. General 4-dimensional Hadamard operators ($H4$) and projectors ($M1$–$M4$) are performed on the bottom three photons. In (**c**), it shows the detection pattern of the four-photon coincidence measurements. The numbers (in blued box) refer to the combinations of the output ports for the four-photon detection, which satisfy the Eq.(15) and return the same quantum controlled operation. The detection pattern shows a success probability of $64/256 = 1/4$. For a $n$-photon $d$-dimensional qudit-based quantum processor, the success probability is thus $\frac{d^{n-1}}{d^n} = \frac{1}{d}$.

multi-qudit entangling state as:

$$|\psi\rangle_c'(n,d) = \frac{1}{\sqrt{d^n}} \sum_{j=0}^{d-1} \sum_{k_1=0}^{d-1} \sum_{k_2=0}^{d-1} ... \sum_{k_{n-1}=0}^{d-1} |j\rangle_1 \otimes \mathcal{O}_{1,j}\mathcal{O}_{2,j}...\mathcal{O}_{n-1,j} |\phi_1\phi_2...\phi_{n-1}\rangle_2 \otimes |k_1k_2...k_{n-1}\rangle_3 \, , j = \{0,1,...d-1\}, \tag{13}$$

where $|j\rangle_1$ presents the control qudit, $|\phi_i\rangle_2$ presents the data qudit, and $|k_i\rangle_3$ presents the layer qudit, all in the logical basis. Performing the $d$-dimensional Hadamard gate on the qudit $|k_i\rangle_3$, and rewriting the qudit $|j\rangle_1$ in the $d$-dimensional Hadamard basis (similar results can be

obtained in the $d$-dimensional Fourier basis, up to some additional phases), it returns a state as:

$$|\psi\rangle_c'(n,d)=\sum_{k_1=0}^{d-1}\sum_{k_2=0}^{d-1}...\sum_{k_{n-1}=0}^{d-1}\frac{\sum_{i=0}^{d-1}|h_i\rangle_1\otimes\sum_{j=0}^{d-1}h_{i,j}h_{j,k_1}h_{j,k_2}...h_{j,k_{n-1}}\mathcal{O}_{1,j}\mathcal{O}_{2,j}...\mathcal{O}_{n-1,j}|\phi_1\phi_2...\phi_{n-1}\rangle_2}{\sqrt{d^{n+1}}}|k_1k_2...k_{n-1}\rangle_3, \tag{14}$$

We consider one case of the quantum controlled operation, when $h_{i,j}h_{j,k_1}h_{j,k_2}...h_{j,k_{n-1}}=1$. That is, $\forall j\in\{0,1,...d-1\}$:

$$(-1)^{i\odot j+j\odot k_1+...j\odot k_{n-1}}=1. \tag{15}$$

It returns $i=k_1\oplus k_2\oplus...\oplus k_{n-1}$, where $\oplus$ denotes the bitwise non-carry addition in the binary representation. One can notice that there are $d^{n-1}$ cases out of $d^n$ cases satisfying this equation. Thus, the success probability of the multiqudit $\mathcal{MVCU}$ gate is a constant value of $1/d$, regardless of the number of photons $n$. As an example, when $d=4$ and $n=4$, Figures S1b and S1c show the diagram of $n$-photon joint measurements ($n$-fold coincidences measurements) and their required output patterns from different combinations of outport ports, that can realise the $n$-photon $d$-QPU with a $1/d$ success probability (given the preparation of the $n$-photon $d$-dimensional GHZ state in advance).

## Supplementary Note 4: Generalised $d$-ary Deutsch's algorithms

The original binary Deutsch-Jozsa algorithm[64] can determine whether a Boolean function $f$: $\{0,1\}^n\rightarrow\{0,1\}$ is either constant or balanced, by a single query of quantum oracle. This can be generalised as the $d$-ary Deutsch-Jozsa algorithm[63], aiming to determine the global property of a multivalue function $f:\{0,1,\dots,d-1\}^n\rightarrow\{0,1,\dots,d-1\}$, whether constant or balanced, by single call of quantum oracle. However it requires $d^{n-1}+1$ queries classically. We define that the function is constant, if $f(x)=f(y)\ \forall x,y\in\{0,1,\dots,d-1\}^n$; otherwise, the function is balanced, if an equal number of the inputs, namely $d^{n-1}$, is mapped to each of the $d$ elements in the codomain. The quantum circuit for implementing the $d$-ary Deutsch-Jozsa algorithm is shown in Fig.4a. Initially, the input state is set as $|0\rangle^{\otimes n}|1\rangle$. Performing the $\mathcal{F}_d$ gate on the $x$-register states, results in a state as

$$\frac{1}{\sqrt{d^n}}\sum_{x=0}^{d^n-1}|x\rangle\otimes\frac{1}{d}\sum_{y=0}^{d-1}\omega^y|y\rangle, \tag{16}$$

where the auxiliary $|x\rangle$-register is a tensor product of $n$-qudits $|x_1,x_2,...x_n\rangle$, and the $|y\rangle$-register loads data to be processed. The key part of the algorithm is the implementation of the $\mathcal{MVCU}$ logic gate where the $\mathcal{U}_f$ is set as $\mathcal{U}_f|x\rangle|y\rangle=|x\rangle|y\oplus f(x)\rangle$. Performing the $\mathcal{MVCU}$ gate yields the state:

$$\frac{1}{\sqrt{d^n}}\sum_{x=0}^{d^n-1}|x\rangle\otimes\frac{1}{\sqrt{d}}\sum_{y=0}^{d-1}\omega^y|y\oplus f(x)\rangle=\frac{1}{\sqrt{d^n}}\sum_{x=0}^{d^n-1}\omega^{-f(x)}|x\rangle\otimes\frac{1}{d}\sum_{y=0}^{d-1}\omega^y|y\rangle. \tag{17}$$

Notably, the global information of function is now all contained in the $x$-register, and now we only need to process the $x$-register state. By executing the $\mathcal{F}_d$ operation on the $x$-register state, we have the output state[63]:

$$\begin{aligned}|\phi\rangle_{\text{output}}&=\frac{1}{d^n}\sum_{j=0}^{d^n-1}\sum_{x=0}^{d^n-1}\omega^{-f(x)}\omega^{j_1x_1\oplus\cdots\oplus j_nx_n}|j_1,\dots,j_n\rangle\\&=\frac{1}{d^n}\sum_{j=0}^{d^n-1}\sum_{x=0}^{d^n-1}\omega^{-f(x)}\omega^{j\cdot x}|j\rangle\end{aligned} \tag{18}$$

Note that the amplitude for the state $|0\rangle^{\otimes n}$ is $\frac{1}{d^n}\sum_{j=0}^{d^n-1}\omega^{-f(x)}$. If $f$ is constant where $f(x)=C\ \forall x$, the amplitude for $|0\rangle^{\otimes n}$ is $\omega^{-C}$ which follows that all the other amplitudes must be zero. That is, for a constant $f$, the $|x\rangle$-register returns the output state:

$$|\phi\rangle_{\text{constant}}=\omega^{-C}|0\rangle^{\otimes n}. \tag{19}$$

If $f$ is balanced, the amplitude for the $|0\rangle^{\otimes n}$ term is $\frac{d^{n-1}}{d^n}\sum_{j=0}^{d-1}\omega^{-j}=0$. Thus, measuring the $x$-register in the computational basis can determine the constant or balance property of the function. That means, if the outcome is zero (all qudits in $x$-register are measured in the $|0\rangle^{\otimes n}$ basis with a unit probability) $f$ is constant, otherwise $f$ is balanced.

Moreover, one can determine explicitly an affine function $f(x_1,\dots,x_n)=A_0\oplus A_1x_1\oplus\cdots\oplus A_nx_n$. This presents the $d$-ary generalisation of Bernstein-Vazirani algorithm[65], which is a variant of the Deutsch-Jozsa algorithm. In this algorithm, the task is to compute the values of

$A_i$, given $f(x)$ is affine. Substituting the affine function into Eq.18, the output state in the $x$-register can be described as:

$$
\begin{aligned}
|\phi\rangle_{\text{affine}} =& \frac{1}{d^n}\omega^{-A_0}\sum_{j=0}^{d^n-1}\sum_{x=0}^{d^n-1}\omega^{(j_1-A_1)x_1\oplus\cdots\oplus(j_n-A_n)x_n}|j_1,\ldots,j_n\rangle \\
=&\omega^{-A_0}\sum_{j=0}^{d^n-1}\Delta(A_1,j_1)\cdots\Delta(A_n,j_n)|j_1,\ldots,j_n\rangle \\
=&\omega^{-A_0}|A_1,\ldots,A_n\rangle,
\end{aligned} \tag{20}
$$

where $\Delta(x,y)$ is the Kronecker delta function. Measuring the $x$-register in the computational basis can deterministically compute the $d$-ary coefficients $A_1,\ldots,A_n$( $A_0$ is lost as a global phase).

## Supplementary Note 5: Generalised $d$-ary Kitaev's phase estimation algorithm

The task of quantum phase estimation algorithm is to compute the eigenvalue $2\pi\phi$ of an unitary $\mathcal{O}$, that staisfies $\mathcal{O}|\psi\rangle = e^{\mathbb{i}2\pi\phi}|\psi\rangle$, given the eigenstate of $|\psi\rangle$ by variational or adiabatic methods[26,66,69]. Quantum circuit for implementing the $d$-ary Kitaev's phase estimation algorithm[26,29,67,69,73] is shown in Fig 5a. The target Hamiltonian is mapped in the unitary $\mathcal{O}$ and its eigenstate is prepared in the $y$-register, while the ancillary $x$-register qudits are measured to return the computational outcomes. The initial qudit state is set as $|0\rangle\otimes|0\rangle^{\otimes n}$. The $y$-register is then prepared in the eigenstate $|\psi\rangle$ of the operator $\mathcal{O}$. Performing the $\mathcal{F}_d$ gate on the $x$-register state results in a superposition state as:

$$
\frac{1}{\sqrt{d}}\sum_{j=0}^{d-1}|j\rangle|\psi\rangle. \tag{21}
$$

The eigenphase $\phi$ can be written in the $d$-ary expansion form as:

$$
\phi=\sum_{i=1}^{m}\frac{\phi_i}{d^i}=0.\phi_1\phi_2\ldots\phi_s\ldots\phi_m000\ldots \tag{22}
$$

where $\phi_s\in\{0,1,\ldots,d-1\}$ refers to the $s$-dit phase value. The eigenphase is thus approximated with an accuracy of $m$-dit. The algorithm is executed by $m$ iterations to obtain the phase $\phi$. The Kitaev's algorithm computes each dit backwardly from the least significant dit from $s=m$ to 1. In the first iteration, *i.e.*, $s=m$, we performed the $d$-ary $\mathcal{MVCU}$ operation with a unitary of $\mathcal{O}^{d^{m-1}}$, and we then obtain the state as:

$$
\frac{1}{\sqrt{d}}\sum_{j=0}^{d-1}e^{\mathbb{i}j2\pi\phi d^{m-1}}|j\rangle|\psi\rangle=\frac{1}{\sqrt{d}}\sum_{j=0}^{d-1}e^{\frac{\mathbb{i}j2\pi\phi_m}{d}}|j\rangle|\psi\rangle. \tag{23}
$$

Applying an inverse $\mathcal{F}^\dagger$ gate on the $x$-register qudit, it returns a compact and remarkable output state as:

$$
|\phi\rangle_{\text{QPEA}}=\frac{1}{d}\sum_{n=0}^{d-1}\sum_{j=0}^{d-1}e^{\frac{\mathbb{i}j2\pi\phi_m}{d}}e^{\frac{-\mathbb{i}j2\pi n}{d}}|n\rangle=\sum_{n=0}^{d-1}\Delta(\phi_m,n)|n\rangle=|\phi_m\rangle. \tag{24}
$$

Therefore, the $m$-th dit of the eignphase $\phi_m$ can be extracted deterministically, with the $d$-ary accuracy, $\phi_m\in\{0,1,\ldots,d-1\}$, by measuring the ancillary $x$-register qudit in the computational basis of $|\phi_m\rangle$. In the following iteration ($s=m-1,m-2,\ldots,1$), the state of the $x$-register qudit after the $\mathcal{MVCU}$ logic gate can be derived as $\frac{1}{\sqrt{d}}\sum_{j=0}^{d-1}e^{\mathbb{i}j2\pi(0.\phi_s\phi_{s+1}\cdots\phi_m)}|j\rangle$. We then implement the $d$-ary Pauli-$\mathcal{Z}_d(\omega_s)$ rotation with an angle of $\omega_s=-0.0\phi_{s+1}\phi_{s+2}\ldots\phi_m$ in the $x$-register. Note the correction rotation is determined by previous measurement outcomes. This process thus returns the output $x$-register state of $|\phi_s\rangle$. In this way, the $d$-ary phase estimation algorithm iteratively computes all $m$ dits of the eigenphase backwardly, where each dit is estimated with the $d$-ary accuracy – resulting in a $\log_2(d)$ improvement of computational accuracy or $\log_2(d)$ speedup of computational time with the same computational precision.

Figure 4 shows the experimental $d$-ary quantum phase estimations of a phase gate $\mathcal{Z}_4$, a generalised Fourier gate $\mathcal{F}_4$ and a random gate $\mathcal{U}_{\text{random}}$. The $\mathcal{U}_{\text{random}}$ is defined in Eq.25.

$$
\mathcal{U}_{random}=\begin{pmatrix}
-0.0504756+0.560784I & -0.594969+0.334087I & -0.15964+0.182145I & -0.2077+0.339956I \\
-0.537513-0.0527596I & 0.345394+0.592134I & -0.428316-0.0545282I & -0.10145-0.204102I \\
0.549949-0.100219I & -0.078668-0.109456I & -0.751179+0.129183I & -0.113824-0.274643I \\
-0.2031+0.193735I & -0.20827-0.0541636I & -0.00971216+0.416962I & 0.697792-0.462647I
\end{pmatrix} \tag{25}
$$

## Supplementary Note 6: Generalised $d$-ary Kitaev's quantum order-finding algorithm

The basis of Shor's factoring algorithm is the quantum order-finding algorithm. The two prime factors of a number $N$ can be found with a high possibility if the order $r$ of $a$ modulo $N$ which is the least positive integer such that $a^r = 1(modN)$ is known[25]. We can find that the quantum order-finding algorithm is just the phase estimation algorithm applied to the unitary

$$\mathcal{U}_a |y\rangle = |ay(modN)\rangle , \tag{26}$$

which has the eigenstate of

$$|\phi_s\rangle = \frac{1}{\sqrt{r}} \sum_{k=0}^{r-1} e^{-\frac{2\pi i sk}{r}} |a^k(modN)\rangle , \tag{27}$$

and $\mathcal{U}_a |\phi_s\rangle = e^{\frac{2\pi i s}{r}} |\phi_s\rangle$. The task is to compute the eigenphase of $s/r$. Since we have no knowledge of $r$, we can't create the eigenstates of $\mathcal{U}_a$. Instead we use the fact that $\frac{1}{\sqrt{r}} \sum_{s=0}^{r-1} |\phi_s\rangle = |1\rangle$ (in decimal representation) and conditionally apply $\mathcal{U}_a$ to the state $|1\rangle$. In prior to applying the $\mathcal{MVCU}$ gate, we prepare a superposition state of $\frac{1}{\sqrt{d^n}} \sum_{j=0}^{d^n-1} |j\rangle |1\rangle$. The $\mathcal{MVCU}$ gate is programmed as

$$\mathcal{MVCU}_a |j\rangle |k\rangle = |j\rangle \mathcal{U}_a^j |k\rangle , \tag{28}$$

which results in the state as

$$\begin{aligned} \mathcal{MVCU}_a \frac{1}{\sqrt{d^n}} \sum_{j=0}^{d^n-1} |j\rangle |1\rangle &= \frac{1}{\sqrt{d^n}} \sum_{j=0}^{d^n-1} |j\rangle |a^j(modN)\rangle \\ &= \frac{1}{\sqrt{r}} \sum_{s=0}^{r-1} \frac{1}{\sqrt{d^n}} \sum_{j=0}^{d^n-1} e^{\frac{2\pi i sj}{r}} |j\rangle |\phi_s\rangle . \end{aligned} \tag{29}$$

Thus, applying the quantum Fourier transform on the $x$-register qudits and measuring the qudits in the computational basis, allow the estimation of $s/r$ with $s$ selected at random.

We here implement Kitaev's quantum order-finding in $d$-ary by recycling the qudit. This approach replaces multiqudit controls in the $x$-register by a single ancillary qudit. See the semi-classical circuit in Fig.4a. We initialised the $y$-register as $|0\rangle$ and applied a multivalue controlled-unitary gate as:

$$\mathcal{MVCO}_a |j\rangle |k\rangle = |j\rangle |k + a^j(modN)\rangle . \tag{30}$$

Implementing the $\mathcal{MVCO}$ gate yields the state of

$$\mathcal{MVCO}_a \frac{1}{\sqrt{d}} \sum_{j=0}^{d-1} |j\rangle |0\rangle = \frac{1}{\sqrt{d}} \sum_{j=0}^{d-1} |j\rangle |a^j(modN)\rangle , \tag{31}$$

which is equivalent to Eq.29. We then implemented the semi-classical $d$-ary Fourier transform to simplify the circuit, in analogue to the qubit-recycling method in Refs.[26–28, 70–72]. Following the same procedures as the $d$-ary phase estimation, we can thus estimate the $s/r$ with a $\log_2(d)$ improvement of computational accuracy or $\log_2(d)$ speedup of computational time with the same precision.

## Supplementary Note 7: Quantum process tomography

Quantum process tomography allows a full characterisation of quantum operations or quantum gates. The task is to determine a completely positive map $\mathcal{E}$:

$$\mathcal{E}(\rho) = \sum_{m,n=1}^{d^2-1} \chi_{mn} \hat{A}_m \rho \hat{A}_n^\dagger, \tag{32}$$

where $\rho$ is an arbitrary input state, and $\hat{A}_m$ is the basis for operators acting on $\rho$. The process matrix $\chi$ completely describes the process $\mathcal{E}$ once the set of $\hat{A}_m$ has been fixed, and it can be reconstructed from a set of quantum state tomographic measurements[61]. In our experiment, the operators $\hat{A}_m (m \in m = 0, \ldots, 255)$ are chosen as the tensor product of the standard Pauli matrices that are denoted as $\{\mathcal{I}_2, \mathcal{X}_2, \mathcal{Y}_2, \mathcal{Z}_2\}$. As an example, we performed the quantum process tomographic measurement for the $\mathcal{MVCX}_d$ gate. We prepared 256 pure states $|\psi_i\rangle = |\psi_x\rangle \otimes |\psi_y\rangle , i = 0, \ldots, 255$ as the input states in the $x, y$-registers, and processed the state in the $\mathcal{MVCX}_d$ gate, where

$$\{|\psi_x\rangle , |\psi_y\rangle\} = \{|a\rangle , \frac{1}{\sqrt{2}}(|a\rangle + |b\rangle), \frac{1}{\sqrt{2}}(|a\rangle + i\,|b\rangle)\}|_{a,b=0,1,2,3,a<b} \tag{33}$$

All of the 256 input states $\rho_i = |\psi_i\rangle\langle\psi_i|$ are linearly independent. We then reconstructed the output density matrix $\mathcal{E}(\rho_i)$ for each $\rho_i$ using the compressed sensing quantum state tomography techniques[60]. Furthermore, each $\mathcal{E}(\rho_i)$ can be expressed as a linear combination of the basis states,

$$\mathcal{E}(\rho_i) = \sum_k \lambda_{ik}\rho_k, \;\; i,k = 0,\ldots,255, \tag{34}$$

where $\lambda_{ik}$ can be calculated by standard linear algebraic. We thus have

$$\hat{A}_m \rho_i \hat{A}_n^\dagger = \sum_k \beta_{ik}^{mn}\rho_k, \;\; i,k,m,n = 0,\ldots,255. \tag{35}$$

The $\beta_{ik}^{mn}$ are complex numbers which can be calculated, given the $\hat{A}_m$ operators and the input states $\rho_i$. Combining the Eq.32, Eq.34, and Eq.35, we have

$$\sum_k \sum_{mn} \chi_{mn}\beta_{ik}^{mn}\rho_k = \sum_k \lambda_{ik}\rho_k, \;\; i,k,m,n = 0,\ldots,255. \tag{36}$$

From the linear independence of the $\rho_k$ it follows that for each $k$,

$$\sum_{mn} \beta_{ik}^{mn}\chi_{mn} = \lambda_{ik}, \;\; i,k,m,n = 0,\ldots,255. \tag{37}$$

This relation is a necessary and sufficient condition. The reconstructed process matrix for the $\mathcal{MVCX}_d$ gate is provided in Fig.3d. Its quantum process fidelity was measured to be 0.952, defined as $\text{Tr}[\chi_0\chi]$ where $\chi_0$ and $\chi$ are the ideal and reconstructed process matrices, respectively.

## Supplementary Note 8: Number of qudit generators, operators and projectors

In our experiment, We reprogrammed the configuration of waveguide circuits of the $d$-QPU to implement different qudit operations and $d$-ary quantum Fourier algorithms. To show the robustness and reliability of our integrated photonics circuit, We here count the number of qudit generators, operators and projectors in all those experiments. We characterised the $\mathcal{MVCX}_d$, $\mathcal{MVCZ}_d$, $\mathcal{MVCH}_d$, and $\mathcal{MVCF}_d$ gates by measuring their true tables in two complementary bases. Each of the true table measurements requires 5*16=80 state generators, 5*16=80 operators, and 16*16*2=512 projectors. In the Deutsch's experiment, we tested 8 different functions, each of which requires 5 state generator, 5 operators, and 2*4=8 projectors. In the phase estimation experiment, we implemented one-digit measurement for the 4 eigenvalues of $\mathcal{Z}_4$ and $\mathcal{F}_4$ gates, and 12-digit measurement for the 4 eigenvalues of the $\mathcal{U}_{random}$ gate, and 2 eigenvalues of the $\mathcal{F}_d^{0.77}$ gates. In each step of phase estimation, it requires 5 state generators, 5 operators, and 2*4=8 projectors. In the order finding algorithm, we implemented 2*(1+4+16)*5=210 state generators, 2*(1+4+16)*5=210 operators, and 2*2*4*(1+4+16)=336 projectors. We implemented over-complete quantum state tomography (5 state generators, 5 operators and 2*4096 projectors) and compressed sensing quantum state tomography (5 state generators, 5 operators and 2*2048 projectors) for two sets of 4-$d$ Bell states and product state (over-complete), and quantum process tomography for the $\mathcal{MVCX}_d$ gate that consists 256 compressed sensing quantum state tomographic measurements. In total, we reprogrammed the $d$-QPU by in total 1,329,518 configurations of the quantum optical waveguide circuits.

## Supplementary Note 9: Analysis of resources and scalability of the multi-qudit quantum processor

We here analyze and count the required physical resources, that is the number of on-chip phase-shifters and classical controls, when scaling up the processor. Figures 2a and 2b show the scheme of multi-qudit quantum processor, that has one qudit in the auxiliary register and $n$ qudits in the data register, and each qudit is encoded in a $d$-dimensional size. Such a multi-qudit processor requires a number of $(n+1)$ single-qudit generators for state preparation, $(nd)$ local single-qudit operators for multi-qudit $\mathcal{MVCU}$ operation, and $(dn+1)$ single-qudit projectors (see Supplementary Figure 1). We have not counted the resources for the generation of qudit GHZ states, neither other passive components such as MMIs for the quantum erasers. We count the number of phase-shifters, the most expensive resources, which are determined by the number of the qudit generators, operators and projectors. As an example, when $d = 8$, Supplementary Figure 2 shows the schemes for an arbitrary single-qudit operator having $2(d-1)$ phase-shifters, and reversely, so as an arbitrary single-qudit projectors[36]. An arbitrary single-qudit operator requires $(d^2 - d)$ phase-shifters[50,59]. Supplementary Figure 3c shows the number of physical resources, i.e., on-chip phase-shifters, when increasing the number of photons and local dimensionality of each qudit. For example, a 10-photon $d$-QPU requires thousands of phase-shifters and their electronic controls. Using the co-integration technology of photonic and electronic circuits in silicon, it allows the control of thousands phase-shifters or even more.

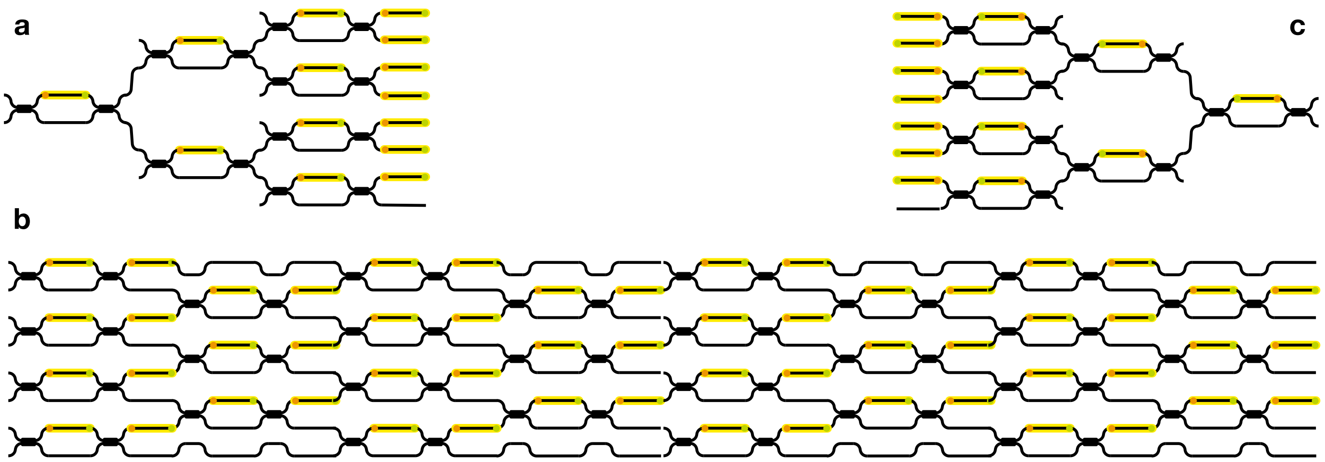

**Supplementary Figure 2: Linear-optical waveguide circuits for single-qudit generator, operator, and projecctor.** **a,** an arbitrary single-qudit generator with $2(d-1)$ phase-shifters, $d = 8$; **b,** an arbitrary single-qudit operator with $d^2 - d$ phase-shifters, $d = 8$; and **c,** an arbitrary single-qudit projector with $2(d-1)$ phase-shifters, $d = 8$.

We now estimate the detection rate of photons, when scaling up the multi-qudit quantum processor. The detection rate is determined by:

$$R_{d-\mathrm{QPU}}(n, d) = R_{\mathrm{GHZ}}(n, d) \cdot \eta_{\mathrm{EOC}}(n, d) \cdot \eta_{\mathrm{MVCU}}(d), \tag{38}$$

where $R_{\mathrm{GHZ}}(n, d)$ presents the rate of qudit GHZ state in the entanglement generation stage I, $\eta_{\mathrm{EOC}}(n, d)$ denotes the attenuation of photons in the "expansion-operation-compression" stage and measurement stage (from stage II to VII, as shown in Fig.2), and $\eta_{\mathrm{MVCU}}(d)$ presents the success probability of the $\mathcal{MVCU}$ gate (that is a constant value of $1/d$, regardless of the number of photons $n$, as discussed above). Both $R_{\mathrm{GHZ}}(n, d)$ and $\eta_{\mathrm{EOC}}(n, d)$ are strongly dependent on the number of photons and the size of each qudit. We first consider the rate of multiphoton multi-dimensional GHZ states[45,46].

$$R_{\mathrm{GHZ}}(n, d) = R_0(\eta_{\mathrm{heralding}}\eta_{\mathrm{OI}}\eta_{\mathrm{SNSPD}})^n p_0^{n/2} \frac{d}{d^n}, \tag{39}$$

where $R_0$ is the repetition rate of a pulsed pump laser (we chose 500MHz), $\eta_{\mathrm{heralding}}$ is the heralding efficiency of photon-pair sources (we chose 0.9 that has been reported in silicon[76]), $\eta_{\mathrm{OI}}$ is the coupling efficiency between silicon chip and optical fiber (we chose 0.89 that has been adopted in our previous experiments[75,77]), $\eta_{\mathrm{SNSPD}}$ is the SNSPD efficiency (we chose 0.9), and $p_0$ denotes the probability of generating one pair of photons, and $\frac{d}{d^n}$ presents the probability of generating the GHZ state from a $n^d$ state (note that other possible post-selections have not been considered in the calculation[45,46]).

The $\eta_{\mathrm{EOC}}(n, d)$ is estimated by:

$$\eta_{\mathrm{EOC}}(n, d) = (\eta_{\text{single-depth}})^{2dn}, \tag{40}$$

where $\eta_{\text{single-depth}}$ denotes the attenuation in a single-depth reconfigurable component, as shown in Supplementary Figure 2, and for each qudit it has $d$-depth in the unitary operator and $(d/2)$-depth in the state generator and projector. We chose $\eta_{\text{single-depth}}$ as 0.98 considering the high-transmission by using directional couplers as on-chip beamsplitters.

One can see the dominator term is $R_{\mathrm{GHZ}}(n, d)$, such that boosting the generation rate of the qudit GHZ state can significantly improve the detection rate of the $d$-QPU. In Supplementary Fig.3d, it shows the estimated detection rate for two cases: case I $p_0 = 0.1$, and case II $p_0 = 0.667$. The case I refers to a typical value of photon-pair generation probability in parametric sources, while the case II is chosen according to the state-of-the-art multiplexing photon-pair source, demonstrated in the bulk-optics system[78]. Combing the state-of-the-art silicon-photonics quantum technologies, a 10-photon multi-qudit quantum processor is achievable in near term. Having high-efficiency multiplexing photon-pair sources, it could reach a 15-photon multi-qudit quantum processor. In long term, it is possible to realise the high-efficiency heralded qudit GHZ states, by using a Fourier transform scheme[31] and advanced multiplexing technologies[78]. In addition, we notice the multi-qudit GHZ states can be generated in the superconducting systems with a high-efficiency[34], which may allow the realisation of multi-qudit $d$-QPU with a high detection rate based on a similar scheme as proposed in Fig.2.

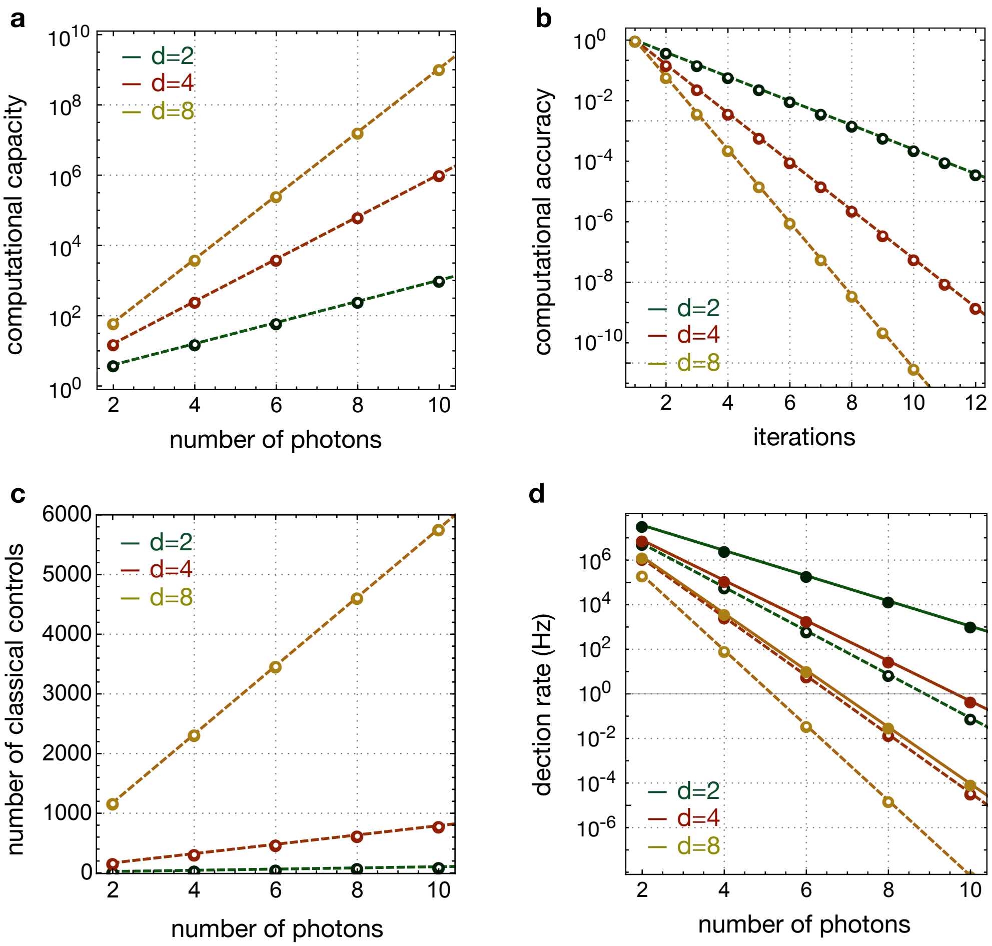


**Supplementary Figure 3: A quantitative analysis of resources and scalability of the multi-qudit quantum processor. a,** The computational capacity is presented by the size of the Hilbert space, having a number of $n$ photons and a local size $d$ of each qudit. That is being said, the capacity exponentially scales with $d^n$. **b,** Enhanced computational accuracy with qudits. As an example, it shows the computational accuracy in the Kitaev's quantum phase estimation for different dimension $d$. It allows a ultimate precision of $\pm d^{-m}$, when perfectly implementing a $m$-iteration phase estimation. To reach the same accuracy, qudit-based quantum computing requires a number of $\log_2(d)$-less iterations, compared with the qubit counterparts. **c,** Calculated physical resources, that is the number of phase-shifters and classical controls, when scaling up the processor. As an example, it shows the required number of phase-shifters for a qudit processor with one qudit in the auxiliary register and $n$ qudits in the data register. It polynomially scale with the number of particles. **d,** Estimated detection rate of photons, when scaling up the processor. The detection rate is given by $R_{\text{GHZ}}(n,d) \cdot \eta_{\text{EOC}}(n,d) \cdot \eta_{\text{MVCU}}(d)$, where $R_{\text{GHZ}}(n,d)$ is the generation rate of the qudit GHZ state, and $\eta_{\text{EOC}}(n,d)$ denotes the attenuation of photons in the "expansion-operation-compression" and measurement stages, and $\eta_{\text{MVCU}}(d)$ presents the success probability of the $\mathcal{MVCU}$ gate that is a constant value of $1/d$. It shows the estimated detection rate for two cases: case I $p_0 = 0.1$ (dashed lines), which refers to a typical value of photon-pair generation probability in parametric sources; case II $p_0 = 0.667$ (solid lines), which is chosen according to the state-of-the-art multiplexing photon-pair source in bulk-optics[78].
